# Evaluation of anxiolytic activity of methanolic extract of *Urtica urens* in a mice model

**DOI:** 10.1186/s12993-015-0063-y

**Published:** 2015-04-24

**Authors:** Zouhra Doukkali, Khalid Taghzouti, EL Houcine Bouidida, Mohamed Nadjmouddine, Yahya Cherrah, Katim Alaoui

**Affiliations:** Laboratory of Pharmacology and Toxicology, Faculty of Medicine and Pharmacy, Mohammed V University, Rabat, Morocco; Laboratory of Animal Physiology, Department of Biology, Faculty of Science, Mohammed V University, Rabat, Morocco; National Laboratory of Drug Control, Directorate of Medicines and Pharmacy, Ministry of Health, Rabat, Morocco

**Keywords:** Anxiety, *Urtica urens*, Rota rod test, Hole board test, Light–dark test, Morocco

## Abstract

**Background:**

The present study was designed to study anxiolytic property of methanolic extracts of *Urtica urens;* an important and commonly used for its medicinal properties belongs to urticaceae family.

**Methods:**

The anxiolytic activity was evaluated with the adult mice by hole board test, and the light–dark box test, and motor coordination with the rota rod test. The efficacy of the plant extract (100–400 mg/kg) was compared with the standard anxiolytic drug diazepam (1 mg/kg i.p.)

**Results:**

The extract increased the time spent in the brightly-lit chamber of the light/dark box, as well as in the number of times the animal crossed from one compartment to the other. Performance on the rota rod was unaffected. In the hole board test, the extract significantly increased both head-dip counts and head-dip duration. *Urtica urens*, in contrast to diazepam, had no effect on locomotion.

**Conclusions:**

These results provides support for anxiolytic activity of *Urtica urens*, in line with its medicinal traditional use, and may also suggest a better side-effect profile of *Urtica urens* relative to diazepam.

## Introduction

Being anxious throughout life has implications not just for subjective wellbeing, but also for physical health and longevity [1]. Anxiety is an unpleasant state of inner turmoil, often accompanied by nervous behavior, somatic complaints and rumination [2], when anxiety becomes excessive, it may be considered as an anxiety disorder, and can critically decrease the quality of life inducing several psychosomatic diseases.

This class of disorders, which includes Agoraphobia, Specific Phobia, Social Anxiety Disorder (Social Phobia), Panic Attack, Separation Anxiety Disorder, and Selective Mutism [3], Anxiety is defined as “a state of intense apprehension, uncertainty, and fear resulting from the anticipation of a threatening event or situation, often to a degree that normal physical and psychological functioning is disrupted” [4], The American Psychiatric Association (APA) purports that each of the Anxiety Disorders share features of fear and anxiety. “Fear is the emotional response to real or perceived threat, whereas anxiety is anticipation of future threat” [3].

Approximately two-thirds of the anxious patients respond to the currently available treatments but the magnitude of improvement is still disappointing, besides, they also produce various systemic side effects and exhibit dependence and tolerance on chronic treatment which now have become a major concern about the use of currently used medicines [5].

Anxiolytic substances mostly belonging to the benzodiazepines group, occupy a prominent post in the ranking of the most utilized drugs of man [6], These Classical benzodiazepines act via the benzodiazepine receptors which are present on the GABAA pentameric complex. The most used compound is diazepam (52% of the studies investigating the action of a benzodiazepine full agonist) [7]. However, the clinical uses of benzodiazepines are limited by their side effects such as psychomotor impairment, sedation, myorelaxation, ataxia, amnesia, potentiation of other central depressant drugs and dependence liability [8,9], there is a need of drug which possesses greater efficacy, lesser undesirable effects with minimum or no tolerance and dependence. Herbs are widely accepted sources of medicine, which play an important role in health care programme worldwide [10]. Hense numerous tradionally used plants exhibit pharmacological properties with great potential for therapeutic applications in the treatment of central nervous system disorders, such as anxiety disorders [11,12]. Also because of the increasing desire of people to use herbal medicines in this study been try to anti-anxiety effect of *Urtica urens* (medicinal plant used in folk medicine in Morocco), researchers in Africa particularly in morocco are exploring the traditional remedies to find a suitable cure for these mind affecting diseases. Moreover, Moroccan climate and favored geographical location have contributed to diversity of medicinal plants.

In Morocco, four species of *Urtica* which belongs to the Urticaceae family are available [13]. *Urtica urens* commonly known as a herbaceous annual plant species of the genus *Urtica*, and has long been known to have tranquillizing effects among the Moroccan people [14]. This Small nettle

(*Urtica urens*) has anti-inflammatory effect [15], nettle and their hybrids or mixtures are recommended for symptomatic treatment of rheumatoid arthritis or osteoarthritis and for increased dieresis [16]. Main constituents identified in the plant material are flavonoids, caffeoyl-esters, caffeic acid, scopoletin (cumarin), sitosterol (−3-O-glucoside), polysaccharides, fatty acids (e.g. 13-hydroxyoctadecatrienoic acid), minerals (herba: up to 20%; leaves: 1–5%) [17,18].

Despite the wide spread use of *Urtica urens* as an anxiolytic, there are no pharmacological data to support this, and therefore we undertook the study to evaluate the anxiolytic potential the methanolic extract of *Urtica urens* by using a battery of appropriate rodent test models.

## Materials and methods

### Animals

Adult Swiss mice (20–30 g) of either sex were used for the study. The animals were acquired from the animal experimental centre of Mohammed V souissi University, Medicine and pharmacy Faculty, Rabat. The animals were maintained in a room with controlled temperature (25 ± 1°C) and lighting (light/dark 12:12 h in polypropylene cages, with food and water ad libitum. Animals were acclimatized to laboratory conditions at least 1 h prior to initiation of experiments. The animals were divided into four groups, each consisting of six mice, implemented in all sets of experiments.

### Plant material

The aerial part of *Urtica urens* was collected in April 2013 from the north of Morocco near the town of Wazzan (Jaaouna el Basra), with assistance of a traditional medical practitioner. The plant was authenticated by botanists of scientific institute Pr. M. Ibn Tatou and Pr. Halim Khammar. A voucher specimen (N° RAB78983) was deposited in the Herbarium of Botany Department of the Scientific Institute of Rabat.

### Preparation of the methanolic extract

The aerial part was dried at room temperature and crushed. 700 g of plant material was extracted with six liter of methanol by maceration at room temperature (25°C) over period of 48 hours. Methanol containing the extract was then filtered through Whatman paper and the solvent was vacuum-distilled at 60°C in a rotary evaporator. The remaining extract was finally dried by desiccator. Final extract was a dark green paste, with 11.92% dry weight. The residue was dissolved in water for final suitable concentrations.

### Drugs

The methanolic extract of *Urtica urens* was suspended in distilled water. Diazepam (ampoule 10 mg/2 ml), pharmacy of Avicenna) was diluted with saline to the required concentration before use. It is well known that benzodiazepines act as anxiolytics at low doses and that they induce sedation and muscle relaxant effects at higher doses [19]. Therefore, we used diazepam (1 mg/kg) as a positive control for anxiolytic-like effects.

### Treatment schedule

Experimental groups of mice were treated orally (p. o.) with methanolic extract of *Urtica urens* at doses of (100–400 mg/kg), whereas control groups received normal saline by the same routes. Diazepam (1 mg/kg) was administered intraperitoneally (i.p.). All drugs were freshly prepared before each experiment. The doses of extracts were calculated to administer 0.25 ml of the suspension of extracts to the mice of 20 g. The trial was carried out 30 min after the treatments. The anxiolytic activity was examined by using the light/dark box test and the hole board test, and motor coordination test assessed with the rota rod test.

### Acute toxicity study

The procedure was followed as per OECD 423 guidelines [20] (OECD/OCDE. 2002). The extract was administered orally at a dose of 2000 mg/kg body weight. Mice were kept under observed for 14 days to register possible mortality; their weights were registered and study their behavioral neurological toxicity.

### Light/dark test

The apparatus consisted of two 20 cm × 10 cm × 14 cm plastic boxes: one light compartment painted white and brightly illuminated and the other was dark painted black and dimly illuminated with red light. The mice were allowed to move from one box to the other through an open door between the two boxes. The illumination in the black compartment was 50 lux, in the white area it was increased to 1000 lux, generated by an extra light source. A mouse was put into the light box facing the hole. The transition between the light and the dark box and time spent in the light box were recorded for 5 min.

### Hole board test

The hole board test [21] was adopted in this test. It is made of gray Perspex. The LETICA board (signo 720; Printer LE 3333) of dimensions 40 cm × 40 cm, contained 16 evenly spaced holes (3 cm diameter and 2.2 cm depth), with in-built infra-red sensors was used for the study. The matt finishing of the upper panel avoids reflections which may alter the animal behavior. An animal was placed in the center of the hole board and allowed to freely explore the apparatus for 5 min. The number of times an animal dipped its head into the holes was automatically counted and recorded by the instrument [22].

### Rota rod test

The effect on motor coordination was assessed using a rota-rod apparatus (LE 8500). Rota rod consisted of a base plant form and aniron rod of 3 cm diameter and 30 cm length, with a non-slippery surface. The rod was divided into four equal sections by three disks. The animals were pre-selected in a training session 24 h before the test, based on their ability to remain on the bar (at 12 rpm) for 2 min, and then allowing four mice to walk on the rod at the speed of 12 rpm at the same time observed over a period of 30, 60, and 90 min. Intervals between the mounting of the animal on the rotating bar and falling off of it were registered automatically as the performance time. Time spent in the apparatus was observed for 5 min duration (300 s).

Apparatus was cleaned thoroughly between trials with water. All behavioral recordings were carried out with the observer blind to the treatment the mice had received.

### Statistical analysis

All results are expressed as mean ± standard error of the mean. The data were analyzed statistically using one way analysis of variance ANOVA, followed by the Tukey Kramer post hoc test for multiple comparisons. P < 0.05 was taken to be statistically significant. Results were presented as tables.

## Results

### Acute toxicity study

Following oral administration methanolic extract of *Urtica urens* at a dose of 2000 mg/kg, P.O., animals were observed for signs of toxicity such as convulsions, hypothermia, hyperactivity, and grooming continuously for 2 h and for mortality up to 24 h after administration of the doses. No toxicity and no significant changes in the body weight were observed between the treated and control group.

### Light/dark test

*Urtica urens* at the dose of 200 mg/kg and diazepam (1 mg/kg) induced a significant increment of the time spent by mice on the illuminated side of the apparatus compared to the respective control group (P <0.05, P <0.01), without significantly affecting other parameters (Table 1).

**Table 1:** **Light/dark test**

**Treatment**	**Dose (mg/kg)**	**Time in the light box**	**No. of transition**
Saline		72.98 ± 18.39	10.5 ± 1.910
Diazepam	1	193.4 ± 17.00**	8.5 ± 1.928
Plant extract	100	120.6 ± 24.03	12.33 ± 3.383
Plant extract	200	156.8 ± 24.23*	12.17 ± 1.682
Plant extract	400	83.62 ± 9.96	16.00 ± 0.577

All values are mean ± SEM (n = 6); *p < 0.05, **p < 0.01 when compared to control. One- way ANOVA, Tukey Kramer post hoc test.

### Hole board test

The dose 200 mg/kg of the plant extract significantly increased the number of head dippings as compared to control animals (Table 2).

**Table 2:** **Hole board test**

**Treatment**	**Dose (mg/kg)**	**Number of head dipping**
Saline		10.50 ± 1.3
Diazepam	1	14.83 ± 1.6*
Plant extract	100	14.33 ± 3.3
Plant extract	200	31.5 ± 2.8***
Plant extract	400	17.20 ± 1.5*

All values are mean ± SEM (n = 6); *p < 0.05, ***p < 0.001 when compared to control. One- way ANOVA, Tukey Kramer post hoc test.

### Rota rod test

The data shows that on average the mice treated with 100, 200 and 400 mg/kg p.o. of the methanolic extract of *Urtica urens* were able to maintain equilibrium on the rotating rod and stayed on longer without falling (Table 3). Whereas diazepam (at 1 mg/kg only) showed a significant decrease in the locomotor score when compared to other groups.

**Table 3:** **Rota rod test**

**Treatment**	**Dose (mg/kg)**	**Time (sec) of animals remained without falling from rod**		
		**30 min**	**60 min**	**90 min**
Saline	1 ml	300	300	300
Diazepam	1	92.33 ± 22,45***	199.8 ± 35,34*	217.5 ± 32.58
Plant extract	100	227 ± 33.87	260,7 ± 18.39	265.2 ± 31.19
Plant extract	200	262.5 ± 22.48	264.8 ± 22.99	277.2 ± 22.83
Plant extract	400	240.2 ± 34.51	270.1 ± 17.25	280.1 ± 19.90

All values are mean ± SEM (n = 6); *p < 0.05, ***p < 0.001 when compared to control. One- way ANOVA, Tukey Kramer post hoc test.

Effect of methanolic extract of *Urtica Urens* and diazepam on motor coordination (rota-rod performance) of mice

## Discussion

In the therapy of anxiety disorder or acute anxiety symptoms, a combination of therapeutic interventions is mostly indicated. Beside a psychotherapeutic approach, anxiolytics are a part of treatment of anxiety [23]. Dysregulation of the GABAergique, serotoninergic, dopaminergic and adrenergic neurosystems have all been implicated in the pathophysiology of anxiety [24]. Benzodiazepines are the most widely prescribed for the last 40 years to treat several forms of anxiety; however, they have prominent side effects such as sedation, myorelaxation, ataxia and amnesia, and can cause pharmacological dependence [25]. Other anti-anxiety medications include antidepressants, buspirone and β -blockers which though effective in many cases, also possess side effects like nausea, light headedness, dizziness, headache, dry mouth, constipation, diarrhea, etc. [26].

Self-administration of herbal medicines was among the most popular of alternative therapies, there is considerable interest in the development of new anxiolytics, new therapies for the treatment of anxiety disorders are necessary, and the study of medicinal plants could provide new therapeutic options [12].

In the current work we examined the anxiolytic effects of methanolic extract of *Urtica urens*, using the light/dark test and the hole board, and to examine motor coordination we used Rota rod test. Furthermore, the effects of *Urtica urens* and diazepam on these animal models were compared to determine whether the behavioral profile *Urtica urens* differed from an established anxiolytic drug.

In the light/dark test, anxiety is generated by the conflict between the tendency to explore and the initial tendency to avoid the unfamiliar [27] and can be evaluated according to the number of transitions in to and the time spent in the light chamber [28,29] where in increase in these parameters is considered to reflect anxiolytic-like properties. Our results showed that the extract (200 mg/kg) increased time spent in the light chamber, suggesting anxiolytic action.

The hole board test is useful for modeling anxiety in animals, in this test an anxiolytic-like state may be reflected by an increase in head –dipping behaviors [30,31]. Our results showed that methanolic extract (200 mg/kg) of *urtica urens* increased the head dipping corroborating the anxiolytic-like effect previously shown in the light- dark test.

Rota rod test a classical animal model used to evaluate peripheral neuromuscular blockade and the motor coordination [32], a deficit in motor coordination would very likely affect performance in the behavioral tests. Our findings showed that *Urtica urens* (100-200 mg/kg), unlike diazepam (1 mg/kg), had no significant effect on motor coordination. Furthermore, the extract didn’t affect motor coordination, is additional evidence of centrally mediated actions and not blockade of neuromuscular system [21,33]. The *Urtica urens* extract showed promising anxiolytic effects without causing any neuromuscular side effects.

## Conclusion

The data presented hereby reinforce the traditional use of *Urtica urens* by Moroccan people to treat anxiety [14]. Despite the wide spread traditional use of *Urtica urens* for treating various disorders there are no reports of scientific evaluation of its anxiolytic activity.

Our study shows that the *Urtica urens* extract had marked effects on the anxiety-related behavioural parameters on exposure to the light/dark test and the hole board in mice.

*Urtica urens* extract causes an “anxiolytic” behaviour comparable with the effects of diazepam.

Future studies will be focused on the neurobiological mechanisms of action and possible interactions of *Urtica urens* with classical neurotransmitters and the phytoconstituent(s) responsible for the observed central effects has to be isolated and identified.
